# Supplementary material for: Abscisic acid increases hydrogen peroxide in mitochondria to facilitate stomatal closure
Source: Plant Physiol. 2022 Dec 27;192(1):469–87. doi: 10.1093/plphys/kiac601 (PMC10152677; doi:10.1093/plphys/kiac601)
Supplement: kiac601_Supplementary_Data [file kiac601_supplementary_data.zip › Postiglione and Muday Supplemental File.pdf]

__Postiglione and Muday Supplemental Figures__

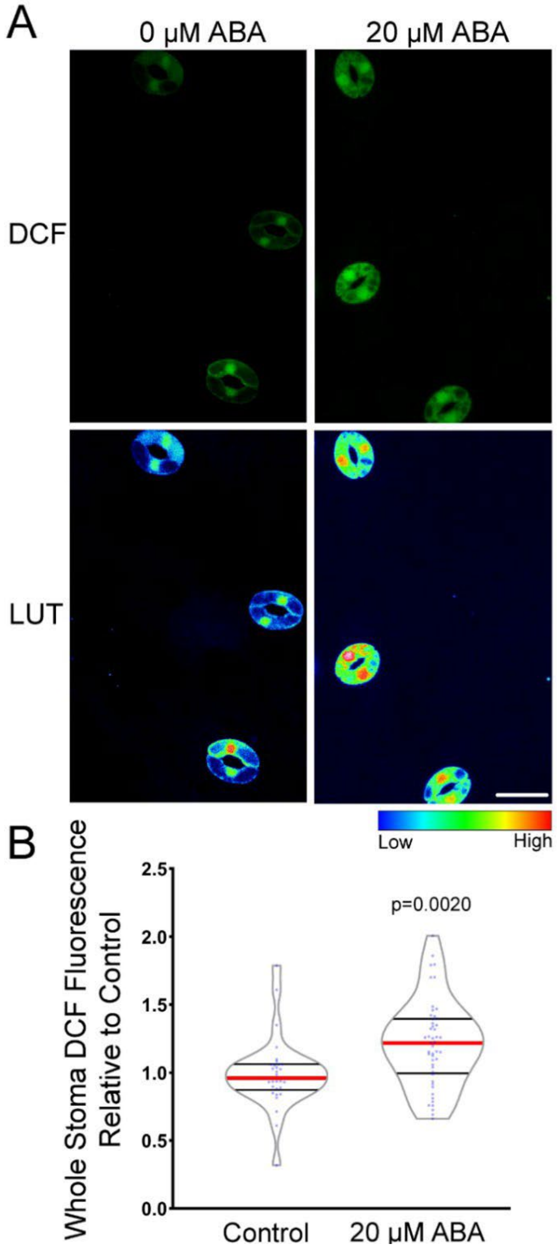

Supplemental Figure S1. Abscisic acid (ABA) increases dichlorofluorescein (DCF) fluorescence when entire Arabidopsis guard cells are quantified.

A) Confocal micrographs of DCF fluorescence of Arabidopsis guard cells treated with buffer control or 20 µM ABA for 45 min shown directly or after conversion to lookup tables (LUT). Scale bar: 20µm. B) Violin plot showing quantifications of DCF fluorescence in the entire guard cell with and without ABA treatment in 30-50 whole stomata per treatment from three separate experiments. All individual values were normalized to the average of the control treatment and are displayed on the graph as blue dots with the median shown in red and lower and upper quartiles indicated in black. *P*-values from paired student t-tests for samples compared to untreated controls are reported.

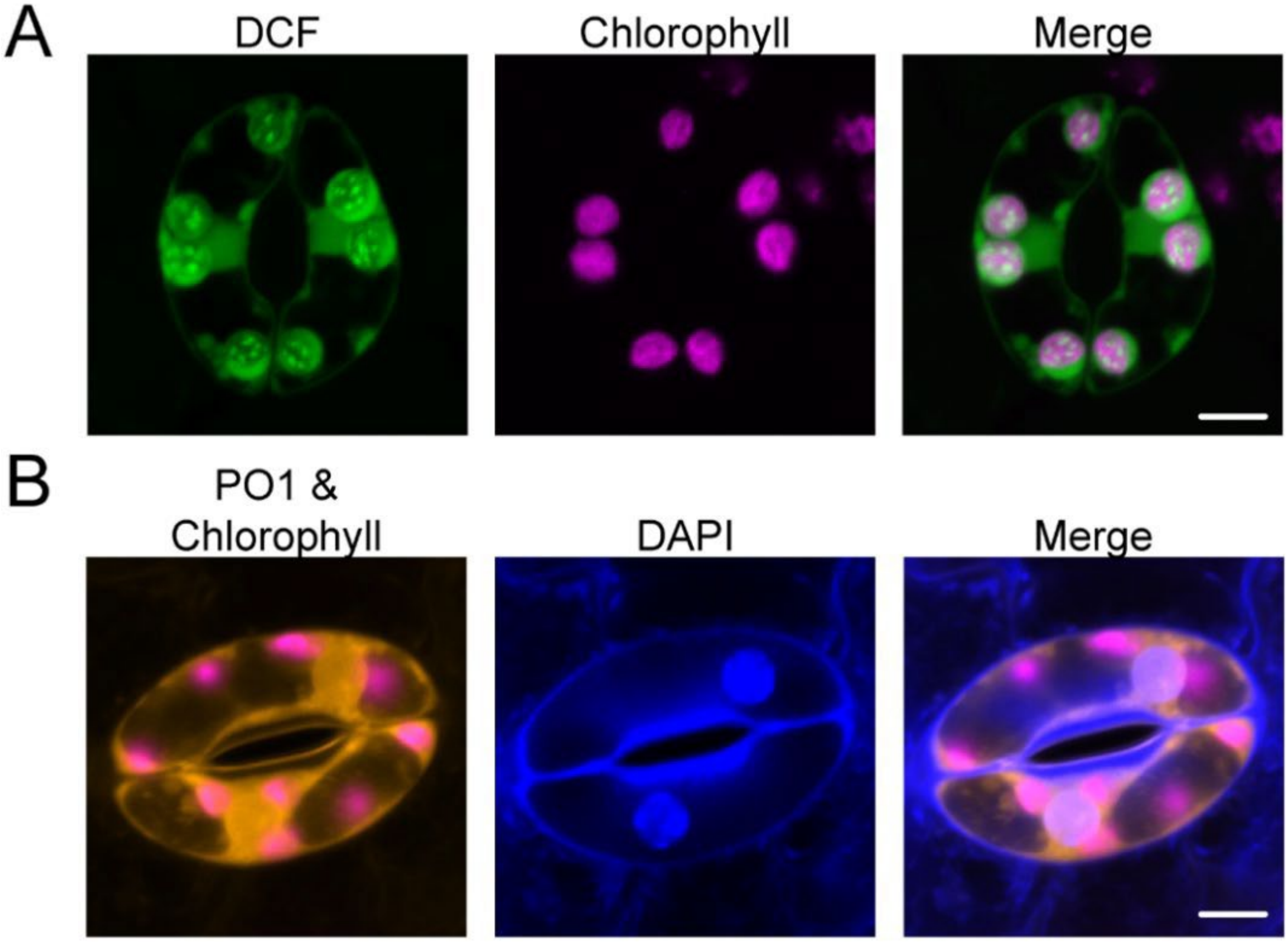

Supplemental Figure S2. Reactive oxygen species-sensitive fluorescent probes accumulate in chloroplasts and nuclei.

A) Confocal micrographs of dichlorofluorescein (DCF) fluorescence (green), chlorophyll autofluorescence (magenta) and merge of both images. B) Confocal micrographs of Peroxy Orange 1 (PO1) (orange) combined with chlorophyll autofluorescence (magenta), DAPI nuclear fluorescence (blue), and a merge of both images. Maximum intensity projections of full z-stacks are shown in for each image. Scale bar: 5µm

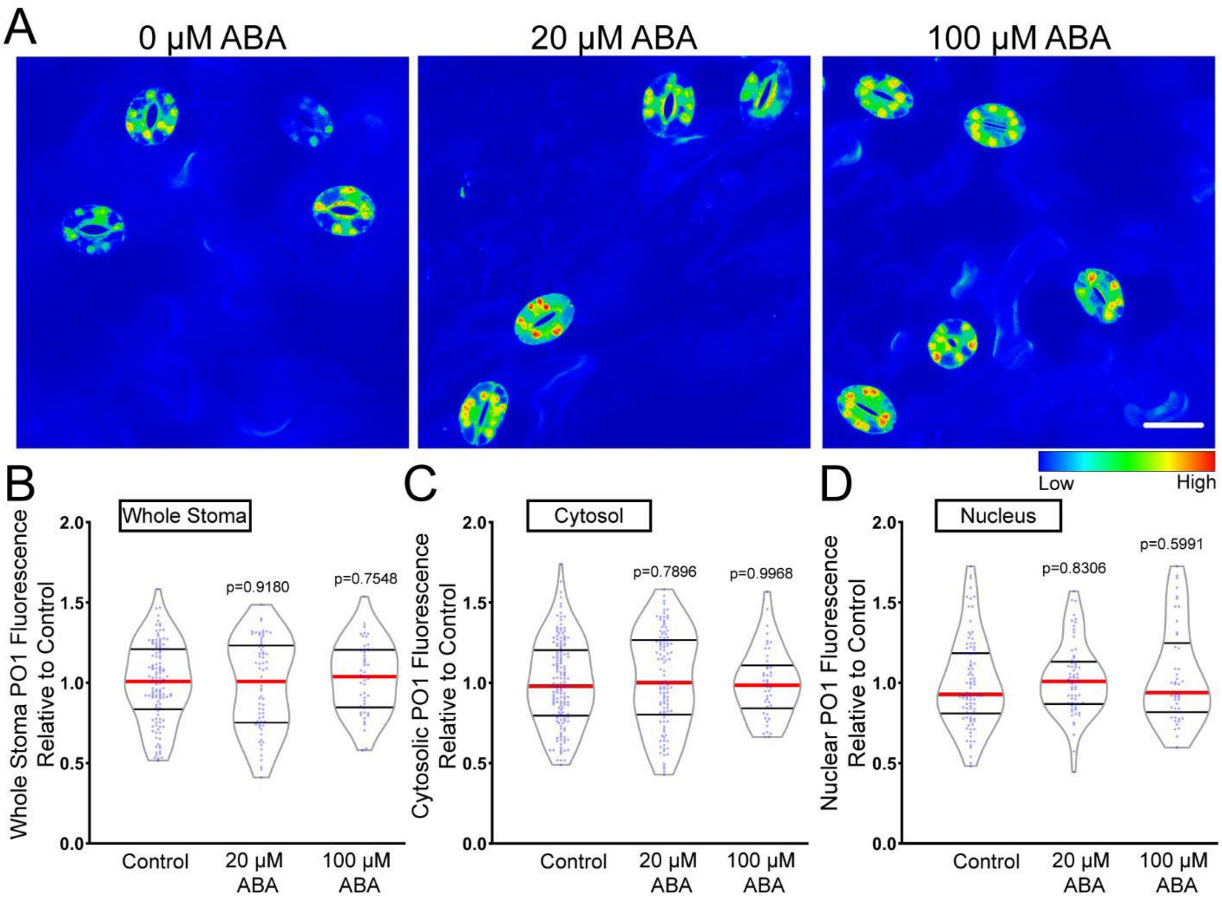

Supplemental Figure S3. There are no significant abscisic acid (ABA)-induced increases in fluorescence of peroxy orange 1 (PO1) in whole stoma, the cytosol, or the nucleus.

A) Confocal micrographs of Peroxy Orange 1 (PO1) signal converted to Lookup Tables (LUT) after treatment with 20 µM or 100 µM ABA or buffer control. Maximum intensity projection of full z-stack is shown. Scale bar: 20µm. Quantifications of PO1 fluorescence in the B) whole stoma, C) cytosol, or D) nucleus. All individual values were normalized to the average of the control treatment for each subcellular location and are displayed on the graph as blue dots with the median shown in red and lower and upper quartiles indicated in black. All *p*-values were calculated by one-way ANOVA followed by a Tukey's multiple comparisons test from at least three separate experiments (chloroplast n>140, nuclear n>50, cytosol n>50, and puncta n>148).

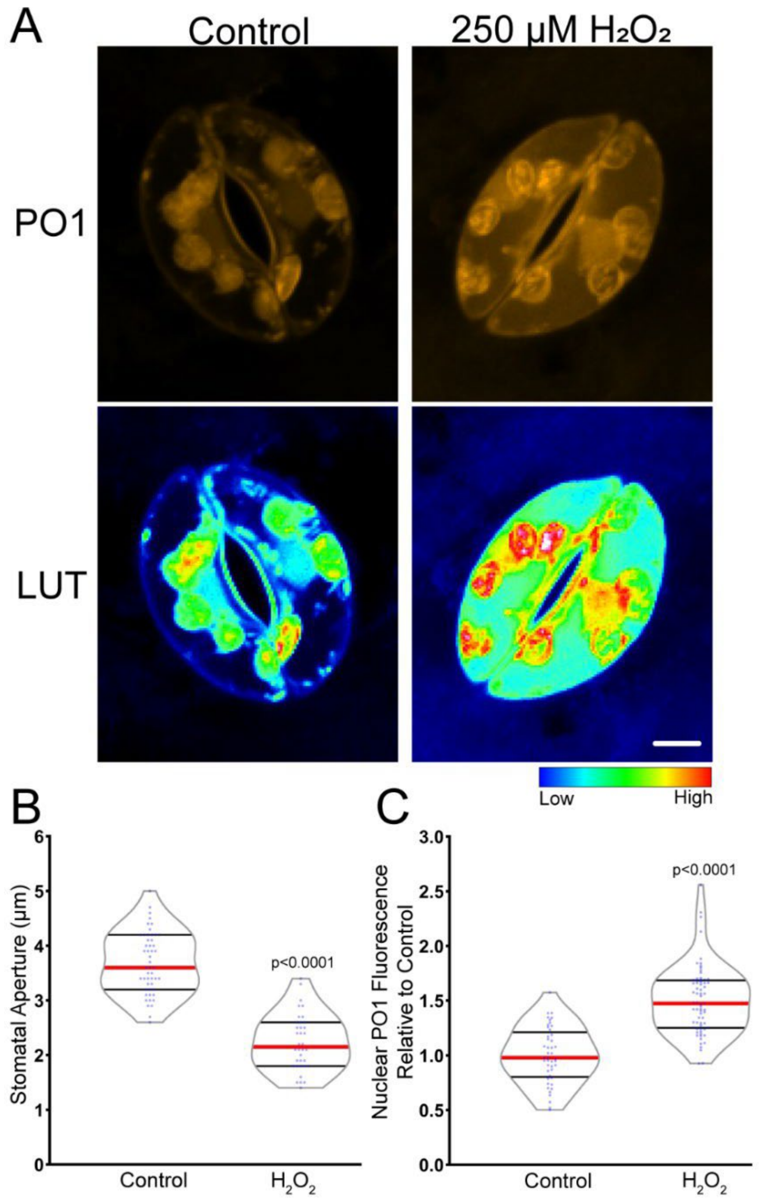

Supplemental Figure S4. Treatment with hydrogen peroxide (H_2O_2) leads to decreased stomatal aperture and elevated Peroxy Orange 1 (PO1) fluorescence.

A) Confocal micrographs of PO1 fluorescence or PO1 fluorescence converted to lookup tables (LUT) of Col-0 guard cells treated with buffer control or 250 µM H_2O_2. Maximum intensity projection of full z-stack is shown. B) Quantification of stomatal aperture with after treatment with either buffer control of 250 µM H_2O_2 C) Quantifications of nuclear PO1 fluorescence with H_2O_2 treatment or buffer control. All values were normalized to the average of the control treatment and are displayed on the graph as blue dots with the median shown in red and lower and upper quartiles indicated in black. *P*-values are recorded according to one-way ANOVA followed by Tukey's post hoc test from at least three separate experiments (n>45). Scale bar: 5µm

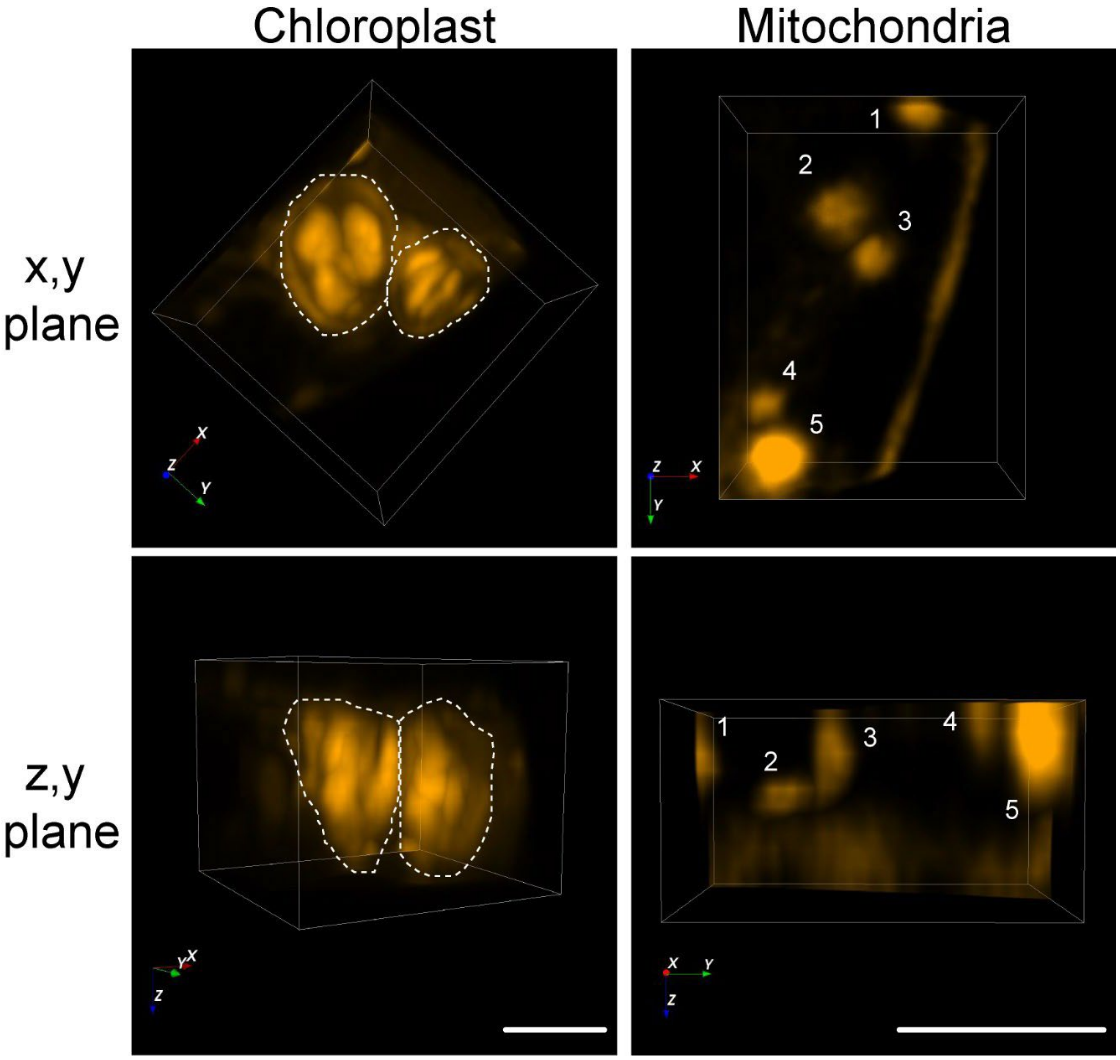

Supplemental Figure S5. Mitochondria have uniform Peroxy Orange 1 (PO1) fluorescence, while chloroplasts have localized regions with elevated signal.

3- dimensional renderings of PO1 fluorescence from a guard cell section displaying either a chloroplast or mitochondria. Renderings are oriented in (x,y) plane or (z,y) plane to show depth. Scale bars: 3µm.

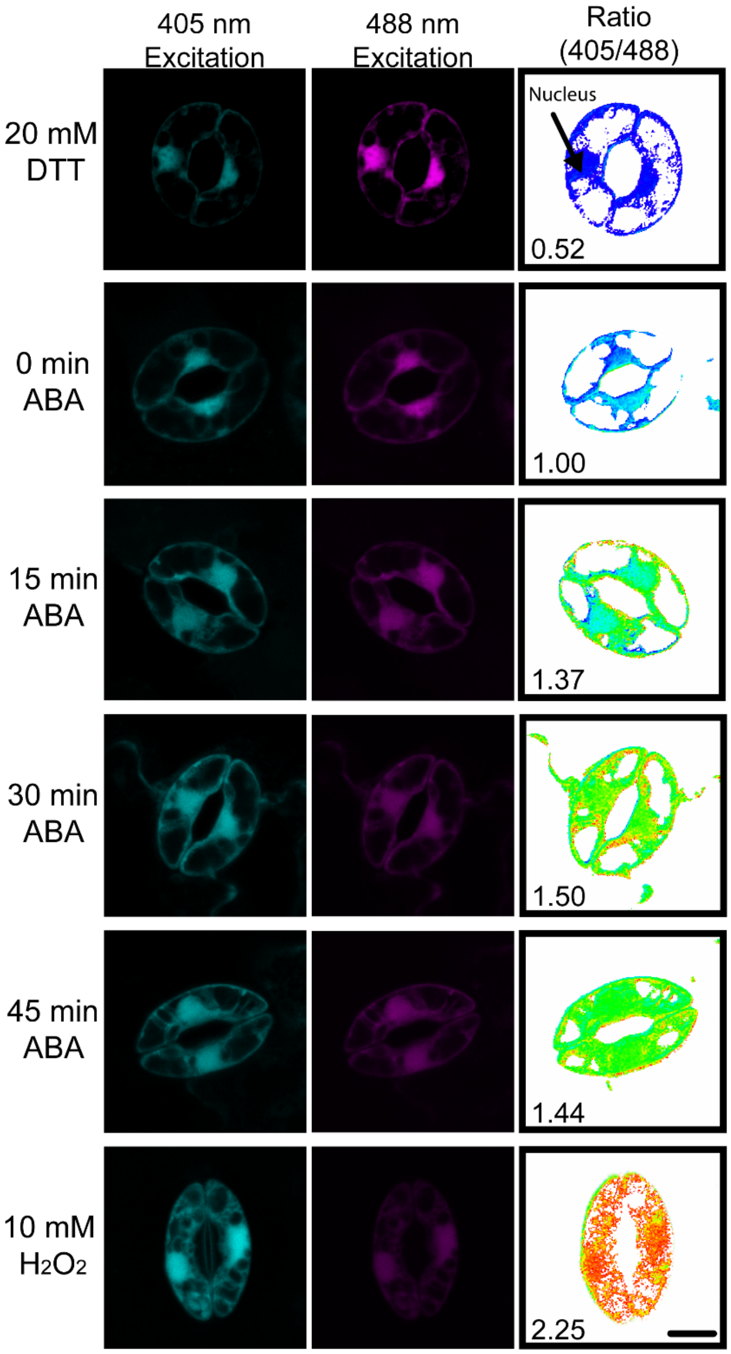

Supplemental Figure S6. Abscisic acid (ABA) treatment alters fluorescence of roGFP2-Orp1 in guard cells consistent with elevated hydrogen peroxide.

Representative micrographs of individual channels used to generate ratiometric images (405 nm/488 nm ratio) seen in Figure 4. Under reducing conditions, signal intensity is high following excitation with the 488 nm laser line (magenta) while excitation with the 405 nm laser lines yields low signal intensity (cyan). As roGFP2- Orp1 becomes oxidized, excitation with the 405 nm laser results in increased signal intensity while excitation with the 488 nm line yields decreased fluorescence. Ratiometric images display fluorescence ratios calculated from images taken using sequential excitation at 488 nm and 405 nm for each treatment group. Scale bars: 5µm.

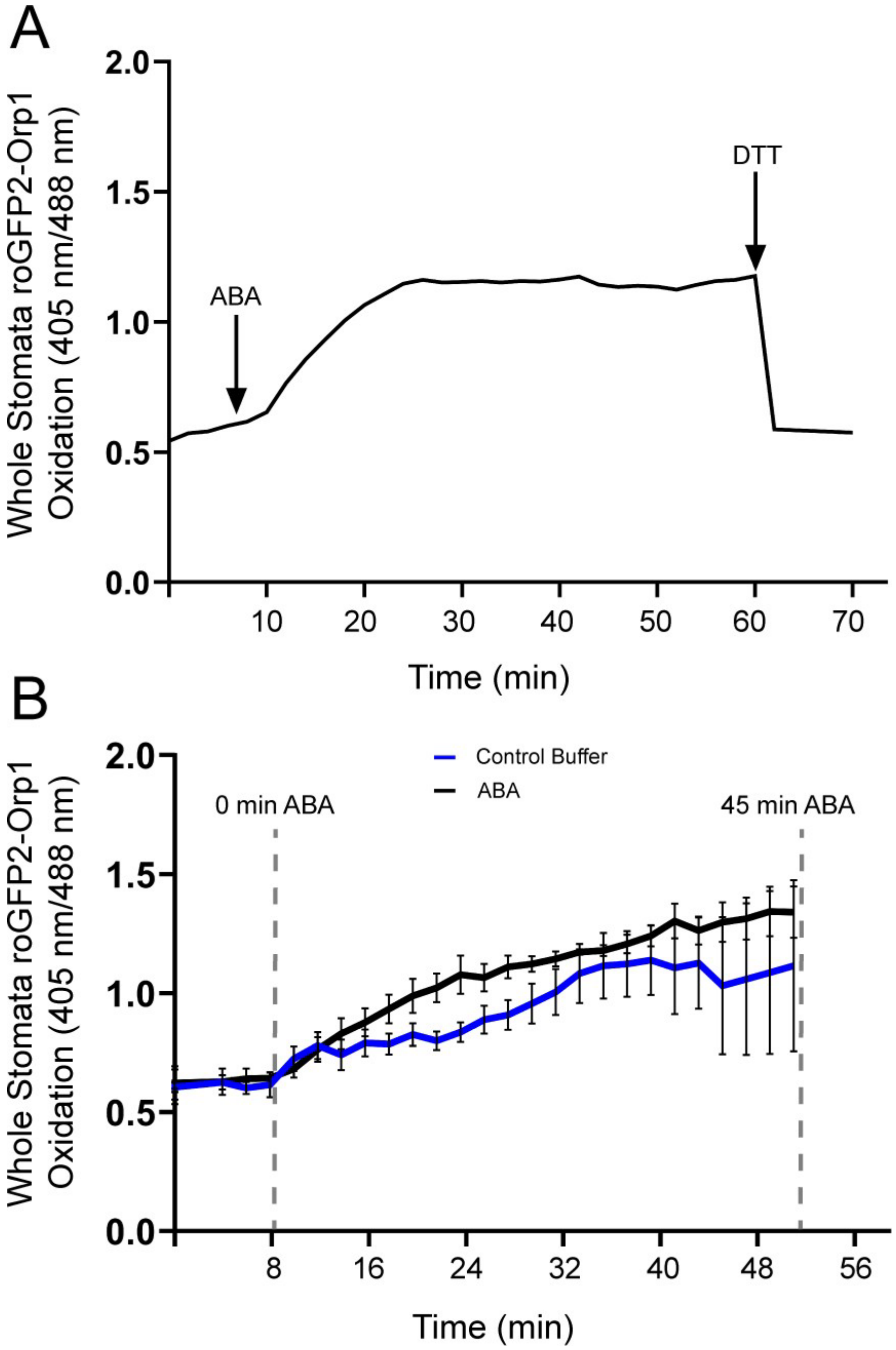

Supplemental Figure S7. Time course imaging of the same subset of roGFP2-Orp1 stomata can result in sensor oxidation even under control conditions.

A) Time course showing abscisic acid (ABA)-driven roGFP2-Orp1 oxidation following treatment with ABA for 50 minutes followed by re-reduction by dithiothreitol DTT. B) Time course showing temporal dynamics of roGFP2-Orp1 oxidation following treatment with ABA (black line) or control buffer (blue line) for 45 min. Stomata were imaged under control conditions for a short period before control buffer was replaced with either ABA or fresh control buffer (first dashed line). Error bars represent SEM. Images were taken every two minutes for the next 45 minutes (second dashed line).

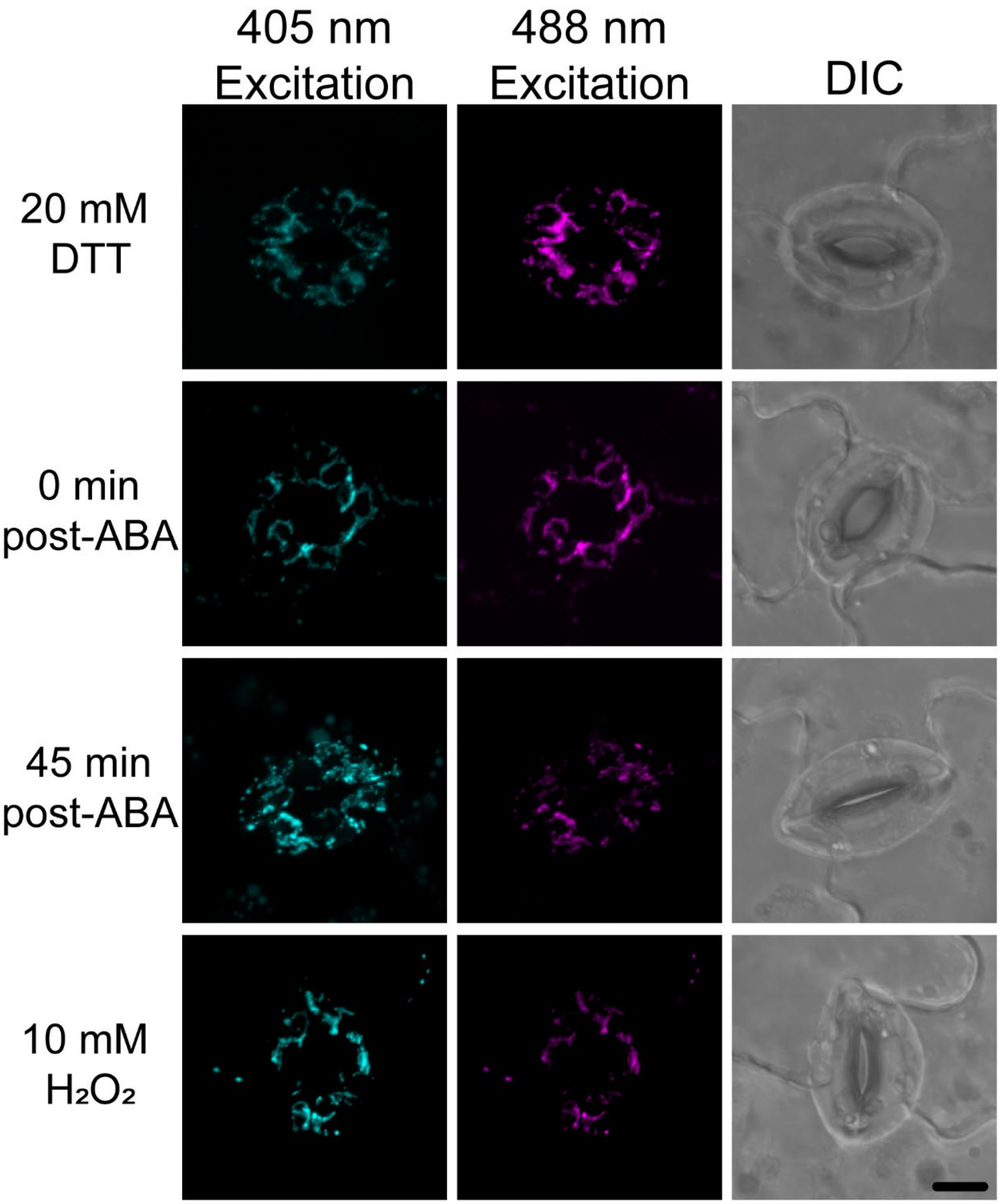

Supplemental Figure S8. A 45-minute abscisic acid (ABA) treatment results in an increased oxidation ratio of mt-roGFP2-Orp1.

Representative micrographs of individual channels used to generate ratiometric images (405 nm/488 nm ratio) seen in Figure 5. Under reducing conditions, signal intensity is high following excitation with the 488 nm laser line (magenta) while excitation with the 405 nm laser lines yields low signal intensity (cyan). As mt-roGFP2-Orp1 becomes oxidized, excitation with the 405 nm laser results in increased signal intensity while signal intensity decreases following excitation with the 488 nm line. Scale bars: 5μm.

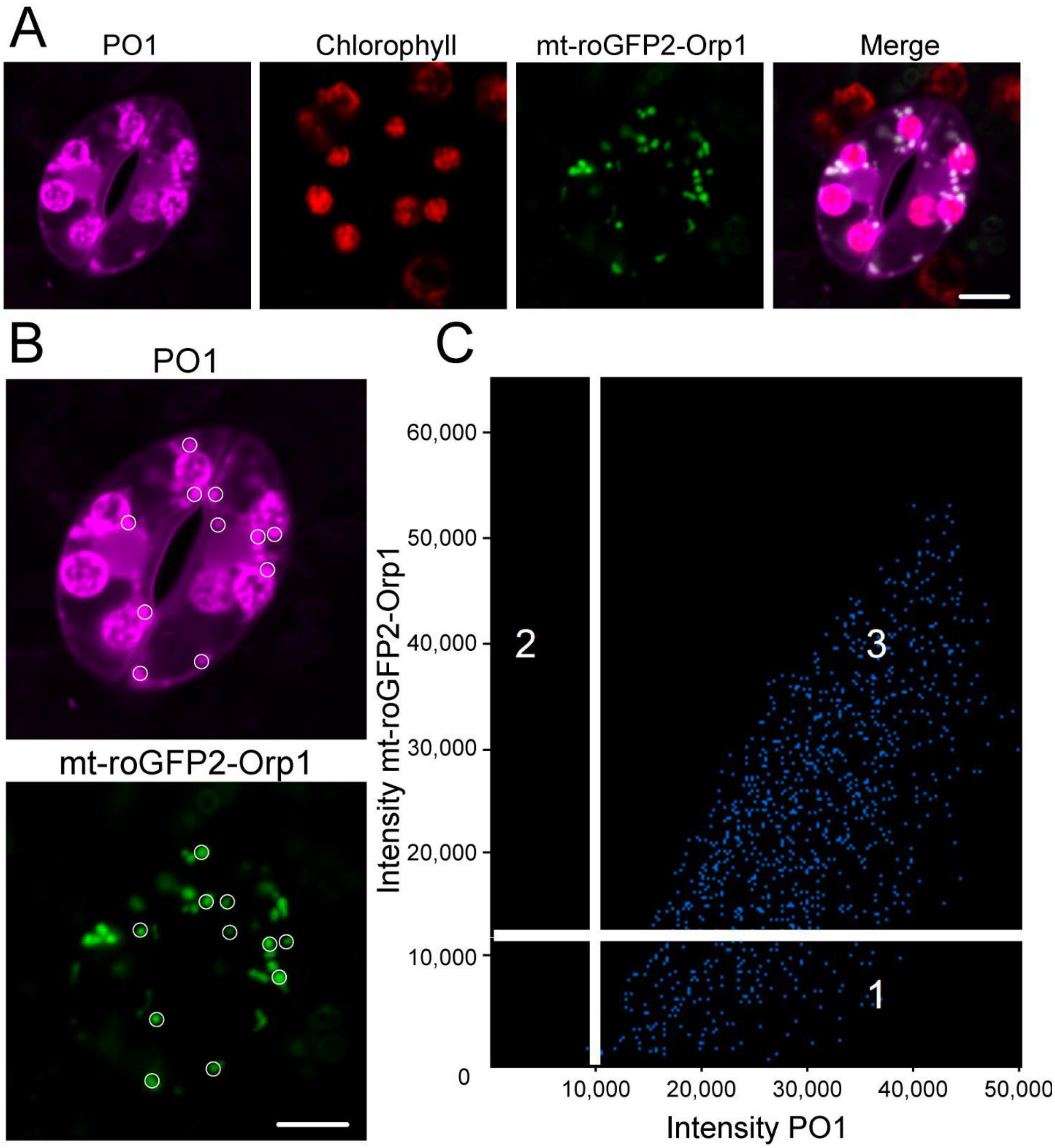

Supplemental Figure S9. Peroxy Orange 1 (PO1) and mt-roGFP2-Orp1 colocalize consistent with abscisic acid-induced reactive oxygen species in mitochondria.

A) Confocal micrographs of PO1 fluorescence (magenta), chlorophyll autofluorescence (red), and mt-roGFP2-Orp1 (green), and a merged image showing PO1 colocalized with mt-roGFP2-Orp1 (white). An individual optical slice is shown. B) Regions of interest used to generate weighted colocalization coefficient are circled in white, highlighting PO1 fluorescence colocalizing with mt-roGFP2-Orp1 fluorescence. C) Colocalization graph generated with the ZEN Black colocalization module from regions of interest highlighting mitochondria. Scale bars: 5µm.

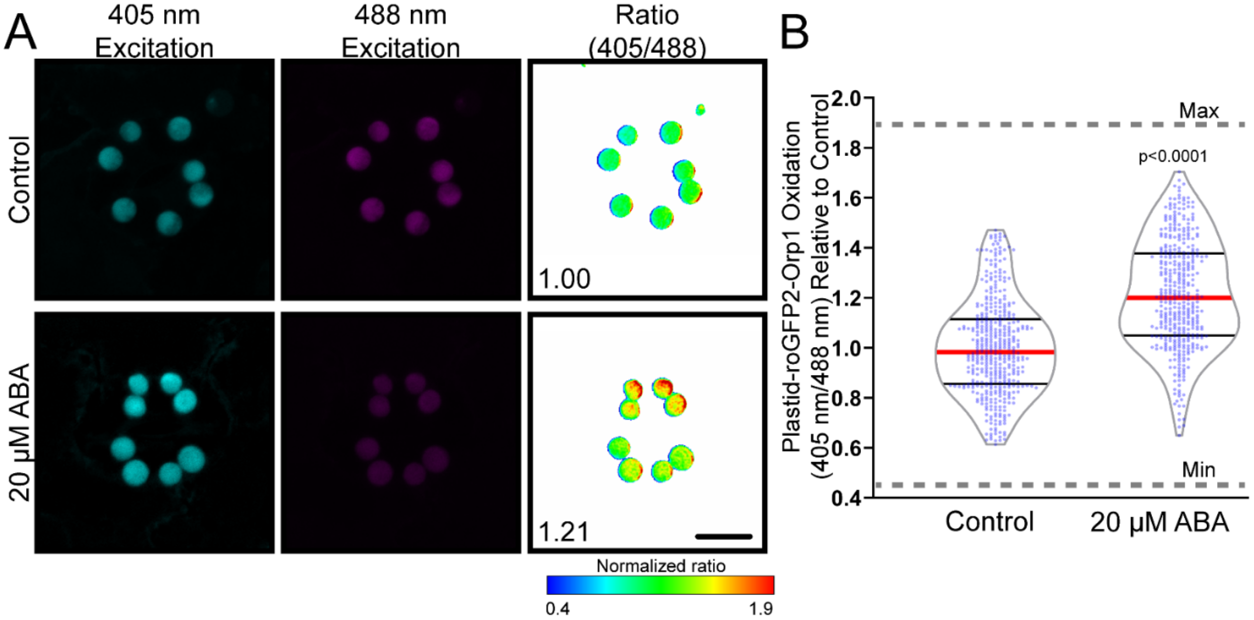

Supplemental Figure S10. Abscisic acid treatment increased oxidation of plastid-roGFP2-Orp1.

A) Representative micrographs of individual channels as well as ratiometric images of oxidation ratio generated through dividing emission intensity follow excitation at 405 nm by emission intensity after excitation at 488 nm. B) Quantification of plastid-roGFP2-Orp1 ratio in the chloroplasts of guard cells following 45 min ABA treatment. All individual values were normalized to the average of the buffer control treated samples and are displayed on the graph as blue dots with the median shown in red and lower and upper quartiles indicated in black. Minimum and maximum sensor oxidation are shown by treatment with 20 mM dithiothreitol (DTT) or 10 mM hydrogen peroxide (H_2O_2), respectively. Data are from three separate experiments (n>400 chloroplasts/value). Minimum and maximum sensor oxidation is represented on graphs by gray dashed lines. Listed *p*-values were determined by one-way ANOVA followed by Tukey's post hoc test. Scale bars: 5µm.

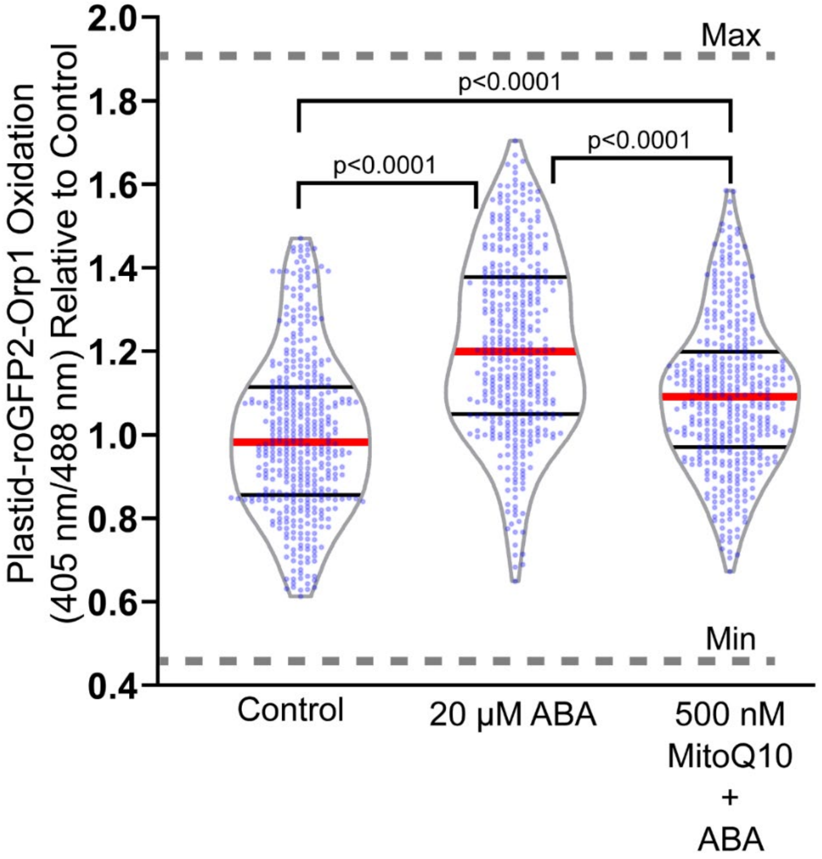

Supplemental Figure S11. The abscisic acid (ABA)-induced oxidation of plastid-roGFP2-Orp1 is reduced by pretreatment with MitoQ.

Quantification of plastid-roGFP2-Orp1 ratio in the chloroplasts of guard cells pretreated with control buffer or 500 nM MitoQ for 3 hours, followed by treatment with 20 μM ABA for 45 min. All individual values were normalized to the average of control treated chloroplasts and are displayed on the graph as blue dots with the median shown in red and lower and upper quartiles indicated in black. Minimum and maximum sensor oxidation are shown by treatment with 20 mM dithiothreitol (DTT) or 10 mM hydrogen peroxide (H2O2), respectively. Data are reported from three separate experiments (n>400 chloroplasts). Minimum and maximum sensor oxidation is represented on graphs by gray dashed lines. Listed *p*-values were determined by one-way ANOVA followed by Tukey's post hoc test. Scale bars: 5μm.

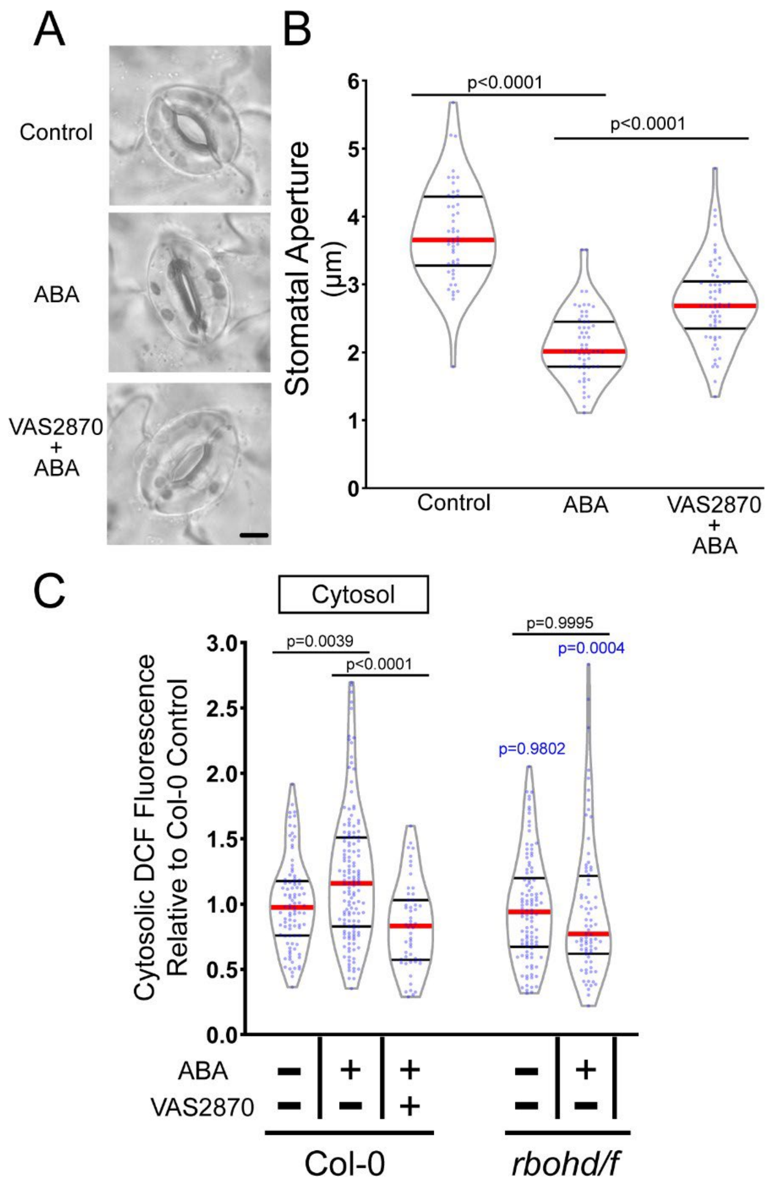

Supplemental Figure S12. The respiratory burst oxidase homolog (RBOH)-selective inhibitor VAS2870 reduces abscisic acid (ABA)-dependent guard cell closure and dichlorofluorescein (DCF) accumulation.

A) Stomatal apertures of leaves of Col-0 treated with a buffer control, ABA, or pre-incubated with 10 µM VAS2870 for 1 hr followed by ABA treatment for 45 min. B) Stomatal apertures of buffer control, ABA, or VAS2870 + ABA were quantified immediately following treatment. C) Violin plots show quantifications of DCF fluorescence in the cytosol following treatment with control buffer, ABA, or pre-treated with VAS2870 and then treated with ABA from three separate experiments (n>52). All individual values were normalized to the average of the control treatment in Col- 0 for each subcellular location and are displayed on the graph as blue dots with the median shown in red and lower and upper quartiles indicated in black. *P*-values in black font represent the significance of differences between treatments in the same genotype spanning the compared treatments. P-values in blue font representing the significance of differences between the indicated genotypes and Col-0. *P*-values are recorded according to two-way ANOVA followed by Tukey’s post hoc test.
